# The Hardening Hypothesis: Further Testing Is Still Required

**DOI:** 10.1093/ntr/ntac125

**Published:** 2022-05-13

**Authors:** Isabella Steffensen, Red Thaddeus D Miguel, Julien Carlone

**Affiliations:** Thera-Business Inc., 515 Legget Dr Suite 204, Kanata, ON, CanadaK2K 3G4; Thera-Business Inc., 515 Legget Dr Suite 204, Kanata, ON, CanadaK2K 3G4; Thera-Business Inc., 515 Legget Dr Suite 204, Kanata, ON, CanadaK2K 3G4

Dear Editor,

The hardening hypothesis suggests that tobacco control activities influence an individual’s ability to quit, and those who find it challenging to quit are those who remain smokers. Reviews and papers have attempted to demystify this hypothesis, and the debate continues. Adding to the continuing discussions, a recently-published umbrella and systematic review by Harris et al. strongly opposed the existence of the hardening hypothesis, after conducting a wide-ranging qualitative synthesis of the evidence pertinent to various hardening constructs and indicators.^1^ However, several key methodological issues may have ushered a deficient—or worse, unqualified—interpretation of the evidence base.

Although it is understandable that systematic reviews do not extract and synthesize all outcomes of studies included around a certain topic, the method is usually guided by a predefined order of measures. However, in the review by Harris et al.^1^, we were surprised that the authors did not include prioritization of outcome measures in their methodology, and included instances where approximate measures were presented over more definitive measures. For example, the review used the study by Edwards et al.^2^ to claim that because smokers’ attitudes to tobacco control measures and goals have softened, their motivation to quit has increased. However, Edwards et al.^2^ also presented results of a more direct measure of motivation to quit, reporting an increase in the proportion of daily smokers who made no attempts to quit lasting more than 24 hours in the past 12 months (although adjusted models found no significant increase); this measure was not discussed at all by Harris et al.^1^

Next, synthesizing repeated cross-sectional studies only assesses changes in individual smoking dependence and quitting measures over multiple timepoints—it is not a reflection of the observed effects and collective process underlying the softening of smokers. By using a single estimate measured through multiple time points, the arguments formed by Harris et al. (2022) were developed under the paradigm of time as the independent variable, and the outcome being change in behavior based on the prevalence of a certain measurement that is cross-sectionally measured. To conclude on the hardening hypothesis based on the mere prevalence of single cross-sectional measures taken at different time points is insufficient. Other evolving measures are needed to better understand the hardening hypothesis—long-term longitudinal studies, for example, could evaluate not just quit rates but also the success of the abstinence made.

Furthermore, Harris et al.^1^ failed to account for alternative causes for the observed softening of smokers by not considering confounders present in the studies. Indeed, in line with the hardening hypothesis that Harris et al.^1^ rejects, a possible explanation for the observed softening could have been an increase in the uptake of harm reduction tools among hardened smokers. The authors acknowledge that only “two studies adjusted for concomitant nicotine administration in the form of snus and nicotine replacement therapy.” Therefore, many of the included studies may have been confounded by the use of harm reduction tools; notably, the need to consider such treatment is not novel and was even discussed in the included Hughes^3^ review. By failing to control for possible confounders, the findings of this review fall short in their ability to attribute the observed trend to any specific cause.

Limiting study designs by cross-sectional studies may also be inadequate to test the hardening hypothesis without considering timepoints of tobacco control activities occurring within the study periods. Additional programs or policy changes are important interventions that could influence conclusions and are overlooked by merely synthesizing studies with a gap of at least five years between the first and last data points. Harris et al. (2022) do not consider multiple pre- and post-intervention effects (eg, authorization of nicotine replacement therapies) that could have been at play throughout the study periods. Studies, like quasi-experimental designs, could have provided a higher level of evidence that accounts for certain policies implemented during the study periods.

Lastly, pertinent information for the interpretation of individual studies synthesized in the review is lacking. One example is the absence of a complete grading system for the risk of bias assessments. Although overall judgments are important, it is as helpful (if not more so) to understand the individual domains around the judgments. Harris et al. (2022) also failed to provide information on the weighting and analytical approaches utilized in the included studies. Often, studies were presented only with their effect estimate, upon which the conclusion was developed. However, repeated cross-sectional surveys could have the propensity for non-response bias, which requires weighting techniques to increase the representativeness of the sample. Additionally, analyzing cross-sectional data longitudinally may take different approaches. Depending on the applied weighting techniques and the way repeated cross-sectional data is analyzed longitudinally, the interpretation of the findings may vary. Thus, to better understand the effect measures, it is important to know how these studies were weighted and analyzed.

On a final note, it is worth mentioning that Harris et al. (2022) report that “All three reviews conclude that hardening has not occurred among the general population of smokers.” Such a definitive statement is not an accurate reflection of two of the three included reviews, which both acknowledge that the hardening hypothesis is occurring in some capacity. Warner and Burns (2003) concluded that hardening is likely occurring in a segment of the population, while also reporting that individuals of lower socioeconomic status—who are part of the general population—are disproportionately represented in this segment.^4^ Although Hughes^3^ report that the data indicate hardening may not be occurring in the general population, their discussion and conclusions are by no means definitive, giving a balanced assessment of possible explanations for their observations, while also recognizing the “paucity of empirical studies” on the hardening hypothesis.

In summary, Harris et al.^1^ exhibit multiple critical methodological issues that may have invalidated the conclusions made by the authors. Until evidence can be synthesized with limited bias, it is difficult to conclude that the hardening hypothesis does not exist.

## Supplementary Material

ntac125_suppl_Supplementary_Taxonomy-form
